# The ELIXIR-EXCELERATE Train-the-Trainer pilot programme: empower researchers to deliver high-quality training

**DOI:** 10.12688/f1000research.12332.1

**Published:** 2017-08-24

**Authors:** Sarah L Morgan, Patricia M Palagi, Pedro L Fernandes, Eija Koperlainen, Jure Dimec, Diana Marek, Lee Larcombe, Gabriella Rustici, Teresa K Attwood, Allegra Via

**Affiliations:** 1 EMBL-European Bioinformatics Institute, Hinxton, Cambridgeshire, CB10 1SD , UK; 2 SIB Swiss Institute of Bioinformatics, Quartier Sorge, Genopode, 1015 Lausanne, Switzerland; 3 Instituto Gulbenkian de Ciência, R. Q.ta Grande 6, 2780-156 Oeiras, Portugal; 4 CSC - IT Center for Science, Keilaranta 14, 02150 Espoo, Finland; 5 Faculty of Medicine , Institute for Biostatistics and Medical Informatics, University of Ljubljana, 1000 Ljubljana, Slovenia; 6 MRC Human Genetics Unit, The Institute of Genetics and Molecular Medicine, University of Edinburgh, Edinburgh, EH4 2XU, UK; 7 Department of Genetics , University of Cambridge, Downing Street, Cambridge , CB2 3EH, UK; 8 School of Computer Science, The University of Manchester, Oxford Road, Manchester , M13 9PL, UK; 9 National Research Council of Italy (CNR), Institute of Molecular Biology and Pathology (IBPM) c/o Department of Biochemical Sciences , Sapienza University, 00185 Rome, Italy

**Keywords:** training, Train-the-Trainer, TtT, instructor training, educational psychology, science of learning, EXCELERATE, ELIXIR

## Abstract

One of the main goals of the ELIXIR-EXCELERATE project from the European Union’s Horizon 2020 programme is to support a pan-European training programme to increase bioinformatics capacity and competency across ELIXIR Nodes. To this end, a Train-the-Trainer (TtT) programme has been developed by the TtT subtask of EXCELERATE’s Training Platform, to try to expose bioinformatics instructors to aspects of pedagogy and evidence-based learning principles, to help them better design, develop and deliver high-quality training in future. As a first step towards such a programme, an ELIXIR-EXCELERATE TtT (EE-TtT) pilot was developed, drawing on existing ‘instructor training’ models, using input both from experienced instructors and from experts in bioinformatics, the cognitive sciences and educational psychology. This manuscript describes the process of defining the pilot programme, illustrates its goals, structure and contents, and discusses its outcomes. From Jan 2016 to Jan 2017, we carried out seven pilot EE-TtT courses (training more than sixty new instructors), collaboratively drafted the training materials, and started establishing a network of trainers and instructors within the ELIXIR community. The EE-TtT pilot represents an essential step towards the development of a sustainable and scalable ELIXIR TtT programme. Indeed, the lessons learned from the pilot, the experience gained, the materials developed, and the analysis of the feedback collected throughout the seven pilot courses have both positioned us to consolidate the programme in the coming years, and contributed to the development of an enthusiastic and expanding ELIXIR community of instructors and trainers.

## Introduction

ELIXIR ( https://www.elixir-europe.org) is a distributed research infrastructure with a mission to manage, provide access to and safeguard the increasing volumes of data being generated by European life scientists. It coordinates and sustains bioinformatics resources across its 21 member states, and helps researchers to more easily find, analyse, share and exchange biological data. As thousands of European scientists make use of ELIXIR’s databases, tools, services and data, providing the necessary bioinformatics training to help them do so most effectively is a key priority, and one of ELIXIR’s main missions ( van Gelder *et al*., 2016).

Bioinformatics seldom forms a core part of formal life-science degree programmes in Europe; therefore, PhD students, postdocs and even PIs seek out focused ‘point of need’ training to gain the skills they require to fruitfully expedite their research. Across Europe, the availability of bioinformatics training opportunities varies greatly from one country to another, and the number of available courses is not sufficient to meet demand, most notably in subjects such as the analysis of next-generation sequencing data. This high demand is not yet waning, and more courses will need to be brought online as the life sciences extend into new areas. Expanding the provision of training in Europe requires the development not only of new courses and materials, but also of new instructors able to deliver high-quality courses. However, the ability to develop new trainers through the provision of ‘instructor training’ is not yet available in all countries.

A continual challenge faced by course providers is finding appropriate individuals to deliver training. Bioinformatics is a dynamic, highly practical and evolving field, and is generally best explained by practitioners in the field. But subject specialists often have little or no formal ‘instructor training’, and hence may be unaware either of learning principles and their application to teaching, of how to tailor sessions to specific audiences, of how to assess whether learning is occurring or has occurred, or of how, ultimately, to design, develop and deliver effective training sessions and materials. 

This situation argues for the development of a practical programme to familiarise subject specialists with good training practices ( Via *et al*., 2013; Best Practices in Training, DEPOCEI Report, 2013, http://www.depocei.org/wp-content/uploads/2013/06/DEPOCEI_Final-report_TRAINING-BEST-PRACTICES.pdf), introducing pedagogical/andragogical theory and methods for course design, delivery and assessment. The idea would not be to try to transform would-be instructors into educationalists, but rather, to help them become responsive, reflective instructors able to provide high-quality training to a variety of audiences, and potentially also able to help others develop their own training skills. Many researcher-instructors (researchers whose career and/or personal interests involve teaching/training) are isolated in their own work settings. Having access to a network or community of practice is therefore important, not just for the support it can provide, but also for enabling their continued development, by sharing ideas and teaching practices, and exploiting training opportunities that arise.

In 2015, ELIXIR received funding for the EXCELERATE project https://www.elixir-europe.org/about-us/how-funded/eu-projects/excelerate) from the European Union’s Horizon 2020 research and innovation programme. One of ELIXIR-EXCELERATE’s main goals is to help ELIXIR support a pan-European training programme to increase bioinformatics capacity and competency.

Against this background, the ELIXIR-EXCELERATE Train-The-Trainer (EE-TtT) programme was developed with the following goals in mind:

1. To assemble research-based training materials using reliable, published sources, and use them to compile an EE-TtT package/framework, to be refined/enriched/reviewed during the programme;
2. To run a pilot of at least six EE-TtT courses in the first twelve months, using the initial EE-TtT package;
3. Deliver a technical workshop to learn from experts about the use of clouds and Virtual Machines (VMs) in training, and develop guidelines for integration into the EE-TtT framework accordingly; and
4. Train further instructor trainers in order to better consolidate the ELIXIR training community, and be able to scale up to several ELIXIR Nodes.

This paper describes the EE-TtT pilot, which was designed and delivered between January 2016 and January 2017. It outlines the focus of the pilot, the content, structure and features of the TtT sessions developed in the first year, and the training materials created so far.

## The EE-TtT pilot overview and goals

The pilot work involved exploring TtT approaches that were already in use within ELIXIR Nodes and related initiatives, and defining a basic framework and curriculum to deliver appropriate training sessions, fit for the needs of the ELIXIR community of bioinformatics instructors ( van Gelder *et al*., 2016). A ‘kick-off’ workshop (see Supplementary Material 1) was held at EMBL-EBI ( http://www.ebi.ac.uk), which brought together representatives from ELIXIR member states with external experts in educational psychology and TtT design and delivery from the Wellcome Trust, NIH-BD2K and Software Carpentry (SWC, https://software-carpentry.org) and Data Carpentry (DC, http://www.datacarpentry.org) Foundations. The objectives of the workshop were to:

• Explore the scope of current activities in the areas of impact assessment and TtT;
• List the methods currently being applied to undertake these activities;
• Identify the major requirements/outputs of each activity;
• Produce a basic framework or set of guidelines for initial development of the TtT programme/pilot; and
• Identify an initial group of candidate TtT trainers.

Workshop participants experienced with bioinformatics training and/or TtT courses or tutoring were invited to elaborate on and present their experiences, and provide five key features a TtT programme should have. The commonalities from the features they provided can be grouped as follows:

1. How TtT sessions should be delivered: they should be interactive and promote sharing of experiences; encourage reflection and development; provide mentoring of new instructors; promote performance videoing and critiquing; provide reference materials for delivering TtT courses; and provide examples of courses to demonstrate possible developments.
2. What TtT sessions should span: they should span pedagogical skills (ability to listen to learners, observe and analyse), and review main evidence-based research results on how learning works; lesson and material planning and preparation with outcomes/aims/learning objectives; effective teaching techniques - practical tips for applying them effectively; evaluation and assessment/feedback by mentor/trainer/learner (including how to handle negative feedback) ( Anderson & Krathwohl, 2001; Bloom & Krathwohl, 1956); identifying skills trainees currently have and what new skills they need to develop and becoming a reflective practitioner.
3. Who should be trained in TtT sessions: the target audience should be current or future committed bioinformatics instructors, in small groups of 20 or fewer participants.
4. What are the challenges of TtT programs: the challenges faced are of sustainability, scalability, building community of practice networks, valuing trainer efforts; continued training opportunities.

A second part of the workshop was dedicated to breakout sessions, inviting all participants to discuss:

• Specific aims of TtT provision across ELIXIR-EXCELERATE;
• Framing an ELIXIR-EXCELERATE TtT course;
• Sources of material for delivery; and
• Who will deliver – how to develop a cohort of TtT trainers.

At the end of the workshop, an outline of the first year of the pilot programme was defined, and individuals to deliver it were identified (intending to train further individuals during the pilot).

In this paper, consistent with the 'instructor' and 'trainer' definitions adopted by the SWC/DC communities, we use the term 'new instructor' to indicate a TtT course completer ('instructor', here, referring to an individual who teaches bioinformatics) and 'trainers' to refer to those individuals who were, or became, qualified to teach EE-TtT courses.

In April 2016, the initial pool of instructor trainers met at the University of Cambridge, UK, to start collecting materials, and to discuss the content and structure of the pilot courses. Two models were particularly influential in this process: the TtT programme at EMBL-EBI ( Watson-Haigh *et al*., 2013) and the SWC/DC Instructor training ( Teal *et al*., 2015; Wilson, 2016). The former inspired the four core topics that were selected for the EE-TtT pilot courses (see Table 1 and the following section). The initial content of each core topic was based on the EMBL-EBI TtT and Carpentries Instructor training materials ( http://swcarpentry.github.io/instructor-training/). The EMBL-EBI TtT is the longest running programme (since 2012) and is focused on providing practical guidance for developing and delivering engaging training in any aspect of bioinformatics, building a network of support for new trainers to exchange ideas and encouraging them to reflect on their practice and development. The teaching philosophy of the SWC/DC communities is based on key research findings about how people learn, and how best to teach them. It was also decided to adopt a number of ‘challenges’ ( *i.e.*, practical exercises); from the Carpentries training we use an exercise in which participants, in groups of three, video each other teaching, then watch the videos and give and act upon feedback. This is combined with exercises from EMBL-EBI training on creating appropriate aims and outcomes, which are peer-reviewed within the group, along with sessions focused on the practicalities of running bioinformatics training. EE-TtT materials are being further developed on the basis of additional exploration of educational psychology principles and theories (see, for example, Ambrose *et al*., 2010; Brown *et al*., 2014; Dunlosky *et al*., 2013; Green, 2015; Lang, 2016)

**Table 1.** Core sessions of the EE-TtT courses. The EE-TtT pilot courses, as well as the training materials, are structured in four main sessions. The four sessions allow for flexibility in the mode of delivery and personalisation from the trainers.

Session #	Element / Subject
Session 1	Principles of learning and how they apply to training
Session 2	Training techniques for enhancing learner engagement and participation
Session 3	Designing engaging sessions, materials and courses
Session 4	Assessment and feedback in training and developing reflective practice

The pilot was delivered in seven EE-TtT courses - each using a slightly different format and focus - and one workshop on ‘Using clouds and virtual machines in bioinformatic training’. Teaching materials were progressively accumulated in a freely accessible GitHub repository ( https://github.com/TrainTheTrainer/EXCELERATE-TtT).

The TtT pilot and training materials are described in more detail below.

## EE-TtT pilot delivery and outcomes

### The EE-TtT pilot training sessions

 As mentioned earlier, the point of the TtT programme is not to produce a new group of educationalists; rather, it is to offer guidance, ideas and tips for training development and delivery based on research-driven educational principles, ultimately aiming to develop a skilled network of instructors able to deliver engaging content in an interactive manner.

During discussions at the kick-off workshop in Cambridge (above), the four main elements listed in Table 1 were identified as the basis for the content and delivery of a course that would meet the original TtT aims.

These four high-level elements were then expanded to create the basis for the content and final structure of the course. The four sessions are built in a flexible / modular manner, so they can be easily delivered according to the needs of the audience. Depending on the type of audience, trainers may decide to emphasise some concepts and activities, while attenuating the relevance of others or even omitting them.

1. **Principles of learning**. This session includes: terms commonly used in the science of learning (albeit often far from standardised); ‘learning objectives’ and ‘learning outcomes’, their differences and uses; how learning works; how memory works; Bloom’s six categories of cognitive skills (knowledge, comprehension, application, analysis, synthesis and evaluation) ( Anderson & Krathwohl, 2001; Bloom & Krathwohl, 1956), how these can generate awareness in teachers, be used to understand the process of learning, and ultimately be applied to develop and deliver a lesson, session or course; most relevant learning theories; learner motivation and demotivation; novices versus experts in learning; the concept of cognitive load; learning methods/activities; active learning and learning-by-doing principles. The seven research-based principles of learning by Ambrose *et al*., 2010, and their implications for teaching practice, are finally listed.
2. **Training techniques**. This session proposes a number of training techniques that have been observed to effectively enhance learners’ engagement and facilitate their learning. Good and bad training practices are explored, and what makes a good teacher/trainer is discussed. In this context, the skills matrix for trainers developed by the TtT taskforce of the Global Organisation for Bioinformatics Learning, Education and Training ( GOBLET http://mygoblet.org, Attwood *et al*., 2015), which provides an overview of the major skills required to be a good trainer, is also introduced. Strategies to motivate learners and promote their engagement, and behaviours that demotivate them are also described, as well as attitudes and activities that can be used to integrate active learning/learning-by-doing strategies in a course. How to create a comfortable and engaging environment for learning is explained; methods for bioinformatics training are also considered.
3. **Designing sessions**. This includes defining the needs of an audience; writing appropriate and realistic learning outcomes using Bloom’s taxonomy; practical methods for lesson design, development and delivery, including the use of learning objectives/outcomes and concept maps; and how to create a concept map and develop a lesson plan. From lesson to session and course design; where to find inspiration. Other related topics include: developing, sharing, archiving and making training materials reusable; training material repositories and resources; training rooms for bioinformatics; reproducibility of training environments; preparatory steps for training delivery.
4. **Assessment and feedback**. This covers formative versus summative assessment; formative assessment in class to gauge trainee engagement/learning; monitoring training impact in real-time; how to use questionnaires to promote peer instruction and content delivery; how to design diagnostic questionnaires to assess learners’ prior knowledge and mental models (multiple choice questions with distractors), and adapt the training accordingly; using learners’ feedback both to assess training quality and instructor performance and as a tool for course development; using feedback as a reflective practitioner; how to gain useful feedback post-course; short- and long-term feedback.

Throughout the course, a balance is struck between theoretical and practical elements, providing an interactive experience where all participants (trainers and instructors) can readily share their thoughts and ideas. All individuals who join the course have some experience of training or teaching - whether it be training / teaching they have delivered, or training / teaching that they have received; this gives everyone a basis for discussion of what training is and is not, or should and should not be. This exchange of thoughts, ideas and experiences makes for a more engaging course, and seeds early network building, and the formation of links that will hopefully endure.

As mentioned previously, the nature of the course, structured in a modular fashion, with an emphasis on interaction and sharing, allows for flexibility in the mode of delivery. This also allows for personalisation from the trainers based on their experience, background and familiarity with learning principles, and the needs, priorities and peculiarities of specific audiences and courses.

During the initial kick-off workshop, two potential models of delivery were identified: stand-alone courses, and courses delivered alongside a training course on a specific subject. These models were implemented in the pilot courses in a variety of different ways ( Table 2), depending on the training opportunities available and the type of learners. The modes of delivery are briefly described below:

**Table 2.** EE-TtT pilot courses. EE-TtT pilot courses were delivered using two main models: 1) standalone courses lasting one or two days; 2) courses including at least one additional session in which learners sit in on a live training event as observers and/or helpers. Afterwards, trainers and trainees sit together to discuss what they saw in the live classroom and what impact that may have on their own practice.

Pilot details	Number of participants	Mode of delivery
Pilot 1: Cambridge, UK 9-10 ^th^ May, 2016	9	2 days – 1 day theory, 1 day practical
Pilot 2: Oeiras, PT 9–15 ^th^ July, 2016	8	6 days – 1 focused on session 1 and 2, 5 run alongside a live training event; session 3 and 4 were delivered at the end of these days together with discussion sessions to critique the live training course in line with TtT participants’ learning.
Pilot 3: Cambridge, UK 11–12 ^th^ July, 2016	11	2 days – 1 day theory, 1 day practical
Pilot 4: Rome, IT 24 ^th^ October, 2016	8	1 day – focus on theory, but with interactive discussions throughout.
Pilot 5: Ljubljana, SL 28–30 ^th^ November, 2016	8	2.5 days - 2 standalone + 0.5 run alongside a live training event.
Pilot 6: Lausanne, CH 16–18 ^th^ January, 2017	7	3 days - 2 standalone + 1 run alongside a live training event.
Pilot 7: Oeiras, PT 19–20 ^th^ January, 2017	10	2 days – standalone

**One-day course**: stand-alone one-day session, focused on providing the theoretical elements listed above, but nevertheless applying an interactive approach ( *i.e.*, ensuring that there is still adequate time for discussion/sharing ideas, *etc*.).

**Two-day course**: the first day focuses on theoretical elements, similar to the one-day course, but day two provides trainees with an opportunity to put into practice some of the elements introduced and discussed in day one. This includes delivering a short ‘training session’ on a topic of their choice and gaining real-time feedback; editing and re-presenting this session to the wider group; working in small groups to expand upon one of their chosen topics to deliver a whole session, including the writing of aims and objectives; drafting requirements to run a course, including computational needs; defining the feedback required both for their practice development and session revision.

**Course alongside training course**: essentially the same as the other modes, but running alongside a training course on a specific subject. The idea is that observing experienced trainers at work, putting elements of pedagogical theory into practice in a ‘live’ setting, and then having the chance to discuss their observations with other trainers is a valuable learning experience. Whenever possible, TtT participants are therefore offered the chance to sit in on a training session on a specific bioinformatics/computational subject (Linux shell, Python, RNA-Seq data analysis, *etc.*) as a supplement to the four core TtT sessions, and time is allocated to discuss the experience.

### The EE-TtT training materials

The structure, content and presentation of the materials was systematically developed as the pilot progressed, and will continue to be honed and reviewed as the project continues.

Materials include items for running the sessions (session outlines, slides, activities, *etc.*), and a set of supporting items, which provide further reading, greater detail, and reinforcement of concepts (references, articles, videos, *etc*.).

**Structure**. The structure of the materials for running the sessions follows the four essential elements listed in Table 1.

**Content**. The content is the result of ideas and materials collected from educational psychology research literature (see, for example, Ambrose *et al.*, 2010; Brown *et al*., 2014; Green, 2015) and from SWC Instructor Training and EMBL-EBI TtT, and also reports a number of successful strategies drawn from trainers’ experiences. Elements of theory are interleaved with activities and practical exercises.

**Presentation**. Materials have been made publicly available through a Github repository ( https://github.com/TrainTheTrainer/EXCELERATE-TtT) and Supplementary file 2, Supplementary file 3. Core materials are currently written in Markdown, and used as visual support in some of the EE-TtT courses. Feedback received at the end of EE-TtT courses in which we displayed content directly from the GitHub website, suggested we should use less distracting and crowded visual aids. We are therefore working to re-organise them into smaller chunks, using a Markdown converter to produce a more interactive format for presentation during courses. Moreover, and importantly, we are working in close collaboration with GOBLET to restructure the materials based on Findable, Accessible, Interoperable and Reusable (FAIR) standards ( Wilkinson *et al*., 2016). A set of slides is also available in the GitHub repository and used as visual support in some courses. 

### Engaging with infrastructures for training set-up

A more technical workshop on using clouds and VMs in bioinformatics training was held at CSC- IT Center for Science (FI) in May 2016, in order to bring everybody abreast with the full potential of these technologies ( https://csc.fi/web/training/-/cloud-vm-bioinformatics). This EE-TtT workshop gathered more than 30 bioinformatics trainers and infrastructure specialists from 13 countries to share their experiences and knowledge on using clouds, VM images and Docker in training.

Bioinformatics analyses typically involve a large amount of software and reference data, making the installation process time-consuming. This problem is aggravated in course settings where every participant needs to have an identical installation, sufficient hardware to run it, and, ideally, access to an identical set-up after the course. VMs and the use of cloud computing can provide solutions to these training challenges. VMs can be run on a laptop, server or cloud (with appropriate virtualisation software), and provide all trainees with an identical software environment in which to work. Additionally, to continue their learning, trainees have the potential to use the VM once a course has ended.

An important element of this workshop was therefore to encourage new instructors to explore and implement the use of VMs and cloud-based systems in their training, and also to provide them with the skills to develop and manage these technologies. The dialogue between bioinformatics instructors and infrastructure providers also allowed the latter to gain valuable knowledge on the special needs that bioinformatics training poses.

All the workshop materials are available in the GitHub ( https://github.com/ekorpela/cloud-vm-workshop/), and the lectures were recorded and made available in YouTube ( https://www.youtube.com/playlist?list=PLD5XtevzF3yHDQZkvO_1kIYd7ZPlmY8j4). Guidelines on how to use clouds, VM images and Docker in training, and how to access computing infrastructures providing these technologies, will be released alongside EE-TtT materials.

## Discussion and future directions

This paper describes the Train-the-Trainer pilot project that was designed, developed and carried out in the context of ELIXIR-EXCELERATE. The EE-TtT pilot represents an essential step towards a sustainable and scalable ELIXIR TtT programme, and the development of a network of trainers and instructors within the ELIXIR community. We ran seven courses, trained more than sixty new instructors and started training three new TtT trainers. We also developed - and have already used - a first draft of supporting materials, and have started structuring them according to the FAIR principles. Most importantly, we have gained further insight into both the potential and challenges of delivering such a programme in the ELIXIR context.

Participant feedback collected at the end of each course has given strong, positive indications of the perceived utility of the programme, but the longer-term impact of the training on the individuals who received it needs to be reviewed. To this end, we will shortly invite all participants to complete a long-term feedback survey, providing us with a more concrete view of the impact this training has had on their own practice. Expectation sessions run during a number of the courses suggested that participants did gain a number of ‘take away’ lessons, and we need to determine whether these were actually put into practice and how this affected the training they delivered.

Another important issue is how to increase the number of TtT trainers who are able to deliver courses independently. The approach taken to date has been to develop interested trainers by having them initially attend a TtT course as a learner, then attend a second course as an assistant. However, the few new candidate TtT trainers who have undertaken this process, despite long-standing experience in bioinformatics training / teaching, felt that, after attending just one or two EE-TtT courses, they were neither knowledgeable nor experienced enough in the pedagogical elements of the course to teach it themselves.

Given that most trainers have had no formal training in education theory, it will be essential to get this balance right, if the programme is to scale. We therefore need to learn from this as the course matures, and capitalise on existing experience. This could include identifying opportunities to work with experienced instructors: for example, becoming a SWC/DC instructor trainer is a quite demanding and compelling process, requiring learners to meet once a week for eight weeks to engage in discussions around teaching pedagogy. New instructors then shadow a teaching demonstration and part of an online instructor training event and attend regular meetings of the trainer community. Their training is completed after delivering two instructor-training workshops (ideally, one in person and one online). To remain an active trainer, a new trainer commits to teach at least two instructor training events per year, among the other things (see https://github.com/carpentries/policies/blob/master/trainer-agreement.md). Furthermore, the availability of very detailed and consistent training materials is an extremely helpful support, especially for new instructors; and the presence of an experienced community of trainers, who meet at least twice a month, is key to sharing experiences and hence developing new trainers’ self-confidence.

Another example from which valuable lessons may be drawn is a recent course run by the EMBL-EBI Node as part of an ongoing collaboration with Australian trainers, in which a group that had previously been participants in a TtT course, with significant training experience, were remotely coached to deliver a very successful TtT session. Their critical feedback will be instrumental in understanding the strengths and weaknesses of the current programme, and the opportunities that may exist to evolve and improve the course, ultimately to create more, and more confident, trainers.

As part of the ongoing, rigorous process of assessment, we put the outcomes of the EE-TtT pilot programme under a microscope, one year after it started. This was done using a Degrees of Freedom Analysis ( Tractenberg, 2017) of the features of the EE-TtT pilot courses, in terms of their utility, feasibility, sustainability of learning, and scalability, with the aim of bringing out strengths and weaknesses of the programme. The results of this detailed analysis are presented in a separate paper ( Via *et al*., 2017).

Thanks to the enthusiastic work of a group of individuals involved in the EE-TtT activities, we designed, developed and reviewed a new TtT pilot programme tailored to the training needs of the ELIXIR community. We are now ready to move forward and consolidate the EE-TtT programme for the remainder of the EXCELERATE period and beyond.

## Supplementary material

**Supplementary file 1** - **EXCELERATE Train-the-trainer and Training Impact workshop report** – This is a document reporting the objectives, agenda, participants, outcomes, and future directions of the EE-TtT ‘kick-off’ workshop described in the manuscript.
