# Supplementary material for: The ELIXIR-EXCELERATE Train-the-Trainer pilot programme: empower researchers to deliver high-quality training
Source: F1000Res. 2017 Aug 24;6:ELIXIR-1557. [Version 1] doi: 10.12688/f1000research.12332.1 (PMC5596339; doi:10.12688/f1000research.12332.1)
Supplement: Supplementary file 1 [file f1000research-6-13350-s0000.tgz › 7ffa2775-97ef-4b21-a50a-13e1d280c1b6.docx]

**
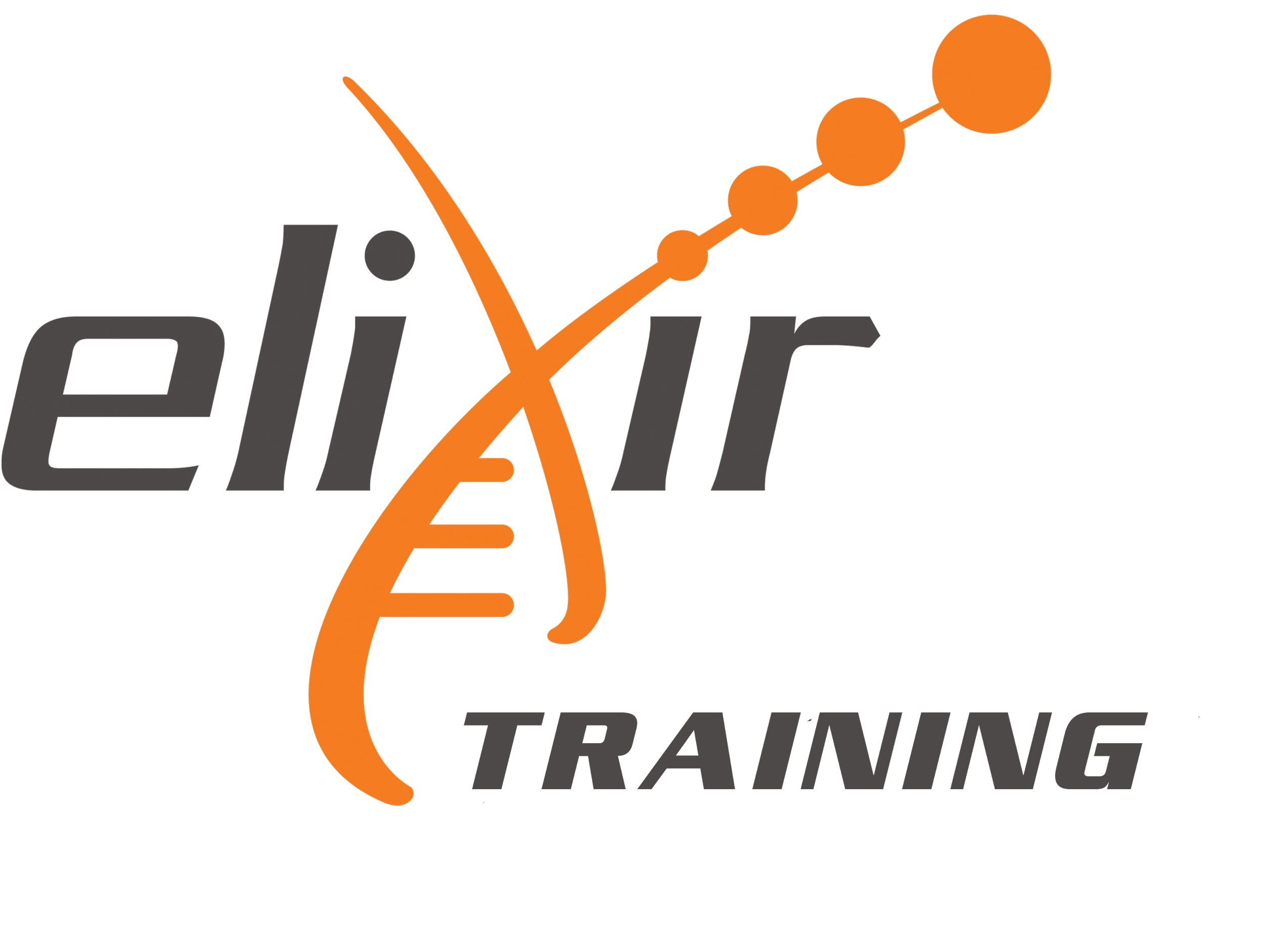
**

**EXCELERATE Train-the-trainer and Training Impact workshop report**

**Gabriella Rustici, Sarah Morgan, Allegra Via**

The EXCELERATE Train-the-trainer (“TtT”) and Training Impact workshop took place on 19^th^-21^st^ January 2016 at EMBL-EBI, Hinxton. The workshop brought together several members of the Training Coordinator’s Group (“TrCG”) as well as representatives of Wellcome Trust Advanced Courses, NIH’s Big Data to Knowledge (“BD2K”) and Software/Data Carpentry (“SC/DC”) to kick-off the activities of the “Train-the-Trainer” and “Quality/Impact” subtasks of the ELIXIR-EXCELERATE training WP11.

The objectives of this workshop were:

- To explore the scope of individual’s / organisation’s current activities in the areas of impact assessment and TtT
- List the methods currently being applied to undertake these activities
- Identify the major requirements / outputs of each activity
- Produce a basic framework or set of guidance for the initial development of such activities

**Agenda**

**January 19^th^ | TtT**

9:30 – 10:00 [Introduction to TtT subtask](https://drive.google.com/open?id=0B5ovJsyGNZ3kWmtmN1ZyQ3BPeEU) AV/SM

10:00 – 11:00 Series of presentations introducing current TtT courses and potential current approaches.

- Sarah Morgan (EMBL-EBI, Elixir-EBI)
- Allegra Via (Elixir-Italy)
- Pedro Fernandes (Gulbenkian Institute; Elixir-Portugal)
- Pamela Black (Wellcome Genome Campus)

11:00 – 11:30 Coffee

11:30 – 12:00 Presentations continued

- Tracey Teal (Software Carpentry)
- Rochelle Tractenberg (Georgetown University, BD2K)

12:00 – 12:30 Discussion

Summarise common themes / approaches identified from the individual presentations.

12:30 – 13:30 Lunch

13:30 – 17:00

Breakout discussions:

- Specific aims of TtT provision across EXCELERATE
- Framing an ELIXIR TtT course / workshop
- Sources of material for delivery
- Who will deliver – how to develop a bigger set of TtT instructors?

**January 20^th^ – TtT / Impact**

9:30 – 11:00 Next steps and pilot year

- Format: what do we try in the pilot, what do we roll out later?
- Who to target (node / trainee)

11:00 – 11:30 Coffee

11:30 – 12:30 Timeline for pilot year and summary – workshop outcomes and actions.

12:30 – 13:30 Lunch

13:30 – 14:00 Introduction to Impact subtask GR/SM

14:00 – 15:00 Series of presentations introducing current methods employed for measuring / reviewing training impact

- Sarah Morgan (EMBL-EBI, Elixir-EBI)
- Gabriella Rustici (Uni of Cambridge, Elixir-UK)
- Patricia Palagi (SIB, Elixir-Switzerland)
- Celia Van Gelder (DTL, Elixir-Netherlands)

15:00 – 15:30 Coffee

15:30 – 16:00 Presentations continued

- Pamela Black (Wellcome Genome Campus)
- Rochelle Tractenberg (Georgetown University, BD2K)

16:00 – 16:30 / 17:00 Discussion

Summarise common themes for impact measure and methods / instruments employed for measuring.

**January 21^st^ - Impact**

9:30 – 11:00 / 11:30 – 12:30 (Coffee will be available at 11:00)

Series of Breakout discussion sessions:

- Who are the stakeholders and why they would like these information?
- What do they need to know?
- What do we need to measure and capture and how?

12:30 – 13:30 Lunch

13:30 – 15:00

Series of breakout discussion sessions on moving towards a framework for Elixir

- How will we collect information – where will it come from?
- Reporting systems – to who?
- Next steps

15:00 – 15:30 Coffee

15:30 – 16:30 Workshop summary, actions and outcomes.

**List of Participants**

Allegra Via (Elixir-Italy)

Cath Brooksbank (EMBL-EBI, Elixir-EBI)

Celia Van Gelder (DTL, Elixir-Netherlands)

Chiara garattini (Intel - Impact subtask only)

Gabriella Rustici (Uni of Cambridge, Elixir-UK)

Jason Williams (CSHL, Impact subtask only)

Laura Emery (EMBL-EBI, Elixir-EBI - TtT subtask only)

Lee Larcombe (Elixir-UK)

Pamela Black (Wellcome Trust Advanced Courses)

Patricia Palagi (SIB, Elixir-Switzerland)

Pedro Fernandes (Gulbenkian Institute; Elixir-Portugal)

Rita Hendricusdottir (Elixir-UK)

Rochelle Tractenberg (Georgetown University, BD2K)

Sarah Morgan (EMBL-EBI, Elixir-EBI)

Terry Atwood (Elixir-UK)

Tracey Teal (Software Carpentry)

Vincenza Colonna (CNR, Italy)

**Impact/quality subtask summary - Gabry/Sarah**

**What information are we currently capturing that could be reused?**

This session started with a short talk by Lee Larcombe, from Elixir-UK, introducing the aims and objectives of this subtask. This was followed by a series of short presentations by participants who have already been involved, to some degree, in the assessment of impact.

All speakers were asked what they/their organisation define as impact. Everybody found this quite difficult but the general consensus was to define training impact as “a measure of how participation in a training course improves someone’s understanding and awareness of a particular domain/topic, leading to change in their research/professional development”.

Each speaker then gave an overview of the metrics/measures that they currently collect about the training activities in which they are involved.

Here is a list of the metrics/measures being collected at different sites:

- quantitative data about the course program/individual event statistics (events / days / participants / geographical spread)
- trainers’ quantitative data - effort involved, number of trainers, hours spent in preparation/delivery/post course
- course ratings - overall organization, course content, balance and level, evaluations of each day of the course
- qualitative data - capturing suggestions for improvements and additional training topics.

Several methods are currently used to capture this data, including:

- Institutional booking systems - primarily for quantitative data about course/participants
- post course feedback online or in paper form - primarily for course ratings, short-term. Online systems include Survey Monkey, Virtual Learning Environments (for undergraduate and master's level courses, VLEs), spreadsheets and Moodle sites for individual courses
- informal dialogue.

The information collected is used primarily for:

- internal use: monitoring of activities, program and further course development, strategic decisions
- reporting to scientific advisory boards, steering groups, management, funders
- compiling annual reports
- communications: newsletters, website, external reports

Long-term feedback (from 6 months to several years) was also highlighted as an area that needed development as few organizations have so far implemented such procedures and there is uncertainty on the best format for such type of survey.

The discussion also touched on how to assure and maintain quality of training. Emphasis was put on the importance of describing courses, and associated course materials, in a consistent manner. It was noted that some organizations have already adopted common formats for course descriptions, aims and learning outcomes which could be easily adopted by other organizations.

**Who are the stakeholders and why they would like these information?**

Extensive discussion focused on who are the stakeholder for whom we want to collect data, what they are likely to be wanting to know and what key performance indicators will allow us to best capture such information.

The outcomes of this initial discussion are presented in the table below.

| **Stakeholder** | **What do they want/need to know? At what level?** |
| --- | --- |
| ELIXIR trainers and volunteers | - Are we providing training which is effective and has impact   - To increase engagement from trainers and trainees   - For personal development / recognition |
| ELIXIR leadership (HoN, SAB, IAB, Hub) | - Is funding being invested appropriately?   ● Is there impact on ELIXIR services? Is training helping drive services’ development/usage?  ● Impact on ELIXIR science/benefit for the organization as a whole |
| ELIXIR users (life scientists, developers, medics, curators, industry, Uni, professional bodies, NGOs,....) | ● accessibility/acquisition of new skills - user perception of accessibility before and after training  ● positive effect on research/practice/collaborations |
| National funders/RCs/policy makers/ministries | ● return on investment  ● who are we reaching? numbers, industry participation, .....  ● impact on research at the national level (number of publications)  ● industry/health services practice engagement - skills transferability to Industry |
| EU funders | ● collaborations between member states/research infrastructures - community building  ● interactions between less and more established countries; skills/knowledge transfer  ● good practice distribution across EU  ● perceived societal impact |
| ELIXIR nodes (and member institutions) | ● similarity with national and ELIXIR leadership  ● benefit of ELIXIR membership (to/from) at national and local level |
| General public, taxpayers | ● better use of resources/increased access   - translation into practice/service enhancement |
| Observers/potential new members | ● membership advantages incl. positive effect on research/practice/collaborations |

**What is ELIXIR training**

The general feeling around the room was that, at this stage, priority should be given to define what is considered “ELIXIR training”, before we can proceed with the impact/quality work.

Participants identified two categories of “ELIXIR training”:

- ELIXIR-Europe level training, which has to be related to an ELIXIR service, or tool, or compute or standard, or use case; and
- ELIXIR-Node level training, where the content will be within the Node remit.

Suggested criteria for labeling training as ELIXIR were:

1. Cover an ELIXIR service, tools, compute, standards, use-case or fulfill an educational need or recognised gap in a specific Node community;
2. Meet a set of quality standards e.g. descriptors (to be defined within EXCELERATE WP11); and
3. Report on specific indicators / metrics to the TrCG (to be defined within EXCELERATE WP11).

This definition of ELIXIR training was presented and discussed at the TrCG meeting which took place during the ELIXIR All Hands meeting in Barcelona, on 7-10 March 2016.

All TrCGs present at this meeting agreed on this definition and that it should now be utilized to identify ELIXIR training activities that should meet a set of quality standards and for which specific indicators and metrics should be reported.

**Initial set of recommendations and a strategy going forward**

Over the next few months we need to start using a coordinated approach to describe training events across the ELIXIR training portfolio and acquiring a consistent set of information about ELIXIR training events.

We here provide an initial sets of: (a) descriptors for training events, (b) quantitative metrics for individual training events; and (c) questions for short-term feedback.

We propose that these should be adopted by all training providers across ELIXIR nodes so that we can start capturing a core set of information about ELIXIR training events in a consistent manner. In this way, we will start capturing information and this will help us to create, refine and expand the EXCELERATE framework of quality indicators and metrics.

1. **Descriptors for training events - basic set**

- Title of course
- Course logistics: start/end dates, venue, organizer/course contact
- Course overview
- Course target audience including prerequisites
- Core syllabus / ELIXIR resources used
- Learning objectives

Additional useful information could include: language in which the course is delivered and a programme (including external URL). These are aligned with the [Bioschemas.org Event specifications](https://github.com/BioSchemas/bioschemas/wiki/Event-Group).

1. **Quantitative metrics for individual training events**

- Number of applicants
- Number of attendees
- Career level
- Country of employment
- Where did you hear about the course?
- Academia/industry/healthcare

1. **Questions for short-term feedback**

| **Type of question** | **Question** | **Choices** |
| --- | --- | --- |
| Multiple choice | Overall rating of the entire course | Poor, Satisfactory, Average, Good Excellent |
| Multiple choice | Please rate each section of the course (*Note to organizer: provide a list of individual sessions*). | Did not attend, Poor, Satisfactory, Average, Good Excellent |
| Open-Ended Response | What part of the course did you like the most? |  |
| Open-Ended Response | What was part of the course would you like the least? |  |
| Multiple choice | The balance of theoretical and practical content across the course was | Too practical, about right, too theoretical |
| Multiple choice | Have you used the resources covered in the course before?  (*Note to organizer: can be tailored to specific ELIXIR resources*). | Never, used other, occasionally, frequently |
| Multiple choice | Will you use the tools/resources covered in the course in your future work?  (*Note to organizer: can be tailored to specific ELIXIR resources*). | Yes, probably, maybe, definitely |
| Multiple choice | Would you recommend this course? | Yes, no, maybe  plus “comments box” |
| Open-Ended Response | What other topics would you like to see covered in future training courses? |  |
| Open-Ended Response | Any other comments? |  |

If you wish to contact participants for long-term feedback, you might want to capture at this stage their name, email address and willingness to be contacted in the future.

**Next steps for each TrCGs:**

1. Identify a couple of upcoming training events taking place in your Node that fulfill the “ELIXIR training” definition;
2. Describe them utilizing the basic set of descriptors in (a) on page 8 of this document;
3. Put in place a mechanism to capture (b) and (c) listed above. A spreadsheet based solution would probably be the easiest way to capture and distribute this for now.
4. We will review your experience with the above in August/September when the Impact Training Coordinator, Louisa Bellis, will start working.
