# Supplementary material for: The ELIXIR-EXCELERATE Train-the-Trainer pilot programme: empower researchers to deliver high-quality training
Source: F1000Res. 2017 Aug 24;6:ELIXIR-1557. [Version 1] doi: 10.12688/f1000research.12332.1 (PMC5596339; doi:10.12688/f1000research.12332.1)
Supplement: Peer review history

# Peer review: Referee response for version 1

**DOI:** 10.5256/f1000research.13350.r25363

**Published:** 2017-09-12

**Authors:** Kim T. Gurwitz

**Affiliations:** 1 H3ABioNet - the pan African bioinformatics network; Computational Biology Division, Department of Integrative Biomedical Sciences, University of Cape Town, Cape Town, South Africa

This is a well-written, clear, and concise article describing the conceptualization, development, and implementation of the Elixir Excelerate train-the-trainer pilot programme (2016-2017). The article highlights best practices for training bioinformatics trainers in how to deliver bioinformatics training to others. The article itself, together with the programme’s freely available materials (links provided within the article), comprise an excellent resource for those interested in learning about, and/or running, a train-the-trainer programme themselves.

 I have 5 additional comments, for consideration:

1. Paragraph 2 and 3 of the Introduction outline the current state of bioinformatics training in Europe. While these statements might be widely accepted in Europe, it is suggested that citations are added, where possible, for a non-European audience.
2. In paragraph 2 of the Introduction, the authors state that ‘Bioinformatics seldom forms a core part of formal life-science degree programmes in Europe’. Perhaps slightly outside the scope of this article, however, it would be interesting to spend one or two more sentences explaining the interplay between formal degree programmes and ‘point of need’ training courses in Europe. In doing so, one might contextualize some of the challenges that bioinformatics trainers encounter as well as the level, and type, of training that they would be expected to deliver against a backdrop of increasing demand for bioinformatics training. Further, it would be interesting to hear the authors’ opinion on why bioinformatics has not been formally incorporated into formal life-science degree programmes in Europe, especially given its increasing importance in the sciences.
3. Page 4 para. 9 – there should be two separate hyperlinks to ‘EMBL-EBI TtT’ and ‘Carpentry Instructors’, respectively. Currently, it is formatted as one long hyperlink with no clear distinction. Further, the full url is only provided for the Carpentries website and not for the EMBL-EBI website.
4. Page 8 para. 6 (‘presentation’) – mentions supplementary files 2 and 3, but links to these are not included at the end of the article?
5. From the text it is apparent that the ‘infrastructure for training set up’ workshop was run as a separate event to the train-the-trainer workshops. Could one argue that infrastructure requirements are integral to teaching bioinformatics as they speak to the learning environment? Perhaps one might consider incorporating an element of this into the core train-the-trainer curriculum, if not already included?

I have read this submission. I believe that I have an appropriate level of expertise to confirm that it is of an acceptable scientific standard.

# Peer review: Referee response for version 1

**DOI:** 10.5256/f1000research.13350.r25365

**Published:** 2017-09-12

**Authors:** Jason J. Williams

**Affiliations:** 1 DNA Learning Center, Cold Spring Harbor Laboratory, Cold Spring Harbor, NY, USA

This article is an important contribution to the global effort of improving bioinformatics training. Nearly everywhere in the life sciences, institutions, laboratories, and individuals are seeking out bioinformatics training and expertise to advance their research. There are lots of solutions, but none have emerged as a clear standard.

 The authors informatively describe the ELIXIR-EXCELERATE train-the-trainers (TtT) pilot as an effort that anyone interested in bioinformatics training will want to pay attention to. The merit of the the ELIXIR TtT approach to not to train on domain science/expertise, but to educate instructors on pedagogical best practices - borrowing from Software/Data Carpentry instructor training and previous EMBL-EBI training efforts.

 The audience trainers will reach is a common one (the biggest? most critical?); researchers who are seeking 'point of need' training. As bioinformatics becomes more skilled at putting tools in researchers hands (Docker, HPC, easy(er)-to-use software), we must also be concerned about users (who may not have bioinformatics/statistical expertise) and their ability to understand the proper use and functionalities of these tools. Importantly therefore, TtT aims to get these domain bioinformatician-trainers to be aware of educational practice - with the goal of helping them better design learning materials and evaluate if they are successfully engaging their audience in learning.

 Overall - the article is very well written. I don't want to change the focus of the report, but I do offer a few comments that might be improvements or prompt the authors to elaborate:

1. On the "commonalities for the features" provided (my page 4 - PDF); Item 1 speaks about "examples of courses to demonstrate possible developments." I was not sure at all what was meant. Should TtT training provide examples of courses where TtT skills are implemented? Something else?
2. (my page 4) you introduce the SWC/DC community trainer definitions. Someone with no knowledge of SWC/DC might not know what this means or why it's important. In the next paragraph you introduce SWC/DC comprehensively, so maybe integrate this with the paragraph below?
3. (my page 5) When you introduce the modes of delivery, I was slightly confused. You said two modes were proposed, but are there three in practice: standalone one-day, stand-alone two-day, and a mixed mode with the one/two-day course held next to a science workshop? Minor, but was confused here.
4. In the Discussion and future directions section (my page 7) you mention the challenges of delivering your program in ELIXIR context? Might you elaborate on one/some of the special challenges of ELIXIR? This would be especially relevant if you could compare to other programs that might have similar challenges in some ways (H3ABioNet Africa?).
5. In the same section as item my 4, you mention SWC/DC instructor training as 8-weeks, they now have a two-day in-person/online version.
6. If I could ask a "follow up" question of this paper, it would be to see some type of summary/profile of the learners who attended the TtT workshops? How many of them had teaching experience? Gender ratios? Just whatever metrics you could provide. I would be curious about how many bioinformaticians that might not see themselves as trainers might see themselves in this cohort.

Thanks for this work.

I have read this submission. I believe that I have an appropriate level of expertise to confirm that it is of an acceptable scientific standard.
